# Design, validity and clinical utility of smartphone app to assess short-term pain trajectories

**DOI:** 10.1186/1745-6215-16-S2-O62

**Published:** 2015-11-16

**Authors:** John Bedson, Kate Dunn, Stephen Dent, Danielle van der Windt

**Affiliations:** 1 Keele University, Stoke-on-Trent, UK

## Objective

Short-term changes in symptoms can be important for accurate diagnosis, predicting long-term outcome (prognosis), and assessing short-term response to treatment. The aim of this project is to design and test a Smartphone application (PainApp) for assessing short-term changes in pain intensity in people with musculoskeletal conditions.

## Methods

The project is carried out in four phases: (I) discussion of objectives, acceptability, and content of the PainApp with Keele’s Research User Group (RUG); (II) design of the PainApp; (III) discussion of face validity and utility with the RUG and clinical advisory group; (IV) testing of acceptability and validity in a prospective cohort study. Primary care consulters (adults, n≥150) with musculoskeletal pain receiving a new prescription for stronger classes of analgesics (opioid combinations, NSAIDs) will be invited to use the PainApp during 4 weeks to enter daily scores for pain intensity, impact of pain on sleep and activities, well-being, analgesics use, and perceived side effects. After 4 weeks, participants will be invited to discuss PainApp recordings with their GP and make further decisions regarding pain management. Questionnaires will be completed at baseline and 1 month follow-up to assess validity of pain recordings and symptom trajectories, and invite participants’ opinions regarding acceptability and usefulness of the App. Medical record review will be used to assess changes in analgesics prescribing over 3 months, compared to a random sample of similar consulters not using the PainApp.

## Results

Phases I - III have been completed, and currently Phase IV is underway.

